# Moral injury among military personnel: a comprehensive review of current research and clinical perspectives

**DOI:** 10.1192/j.eurpsy.2026.12005

**Published:** 2026-07-31

**Authors:** A.-L. Comșa

**Affiliations:** Psychology and Educational Sciences, Babes-Bolyai University, Cluj-Napoca , Romania

## Abstract

**Introduction:**

Moral injury, increasingly recognized alongside posttraumatic stress disorder, refers to the psychological, emotional, and sometimes spiritual distress resulting from actions or experiences that violate deeply held moral beliefs. Clinicians working with morally injured individuals encounter difficulties rooted in both patient needs and their own emotional labour. Addressing the methodological inconsistencies of the studies to date will be crucial if future work is to guide cohesive prevention frameworks capable of mitigating harm before moral injuries solidify into enduring scars on individual identities and collective military communities alike.

**Objectives:**

This study aims to examine the distribution of moral injury research across different military populations over time, identify the measures used for its assessment, and review the intervention strategies that have been applied to date.

**Methods:**

A total of 82 studies met the inclusion criteria. The search was conducted exclusively in PubMed, which provided a comprehensive sample of the literature on moral injury in military populations over the past decade, while keeping the scope manageable for in-depth analysis.

**Results:**

Available evidence suggests that prevalence of moral injury is not evenly distributed across different military branches, reflecting variations in operational environments, exposure risks, and institutional cultures. Operational deployments expose service members to a spectrum of situations that may set the stage for moral injury, and patterns in these exposures are far from uniform. Furthermore, at the individual level, vulnerability to moral injury appears to hinge on a combination of emotional, cognitive, and psychological capacities that shape how potentially morally injurious events (PMIEs) are appraised and integrated into one’s sense of self. Broader societal and cultural contexts exert considerable influence on how moral injury is experienced, interpreted, and addressed within military populations. Current psychotherapeutic interventions addressing moral injury in military populations draw from both established trauma treatments and emerging multidimensional therapies developed specifically to target the moral emotional sequelae of potentially morally injurious events (PMIEs).

**Conclusions:**

Research to date highlights the multifaceted nature of moral injury, involving individual emotional, cognitive, and coping processes, as well as organizational, cultural, and societal influences. The interaction between personal vulnerabilities and structural factors, such as leadership ethics and military culture, illustrates the complexity of addressing moral injury. Emerging models advocate for complex frameworks incorporating psychological, social, spiritual, and biological factors to support moral integrity, healing, and the long-term well-being of military communities.

**Disclosure of Interest:**

None Declared

